# Association of Leukemia With ABO Blood Group Distribution and Discrepancy: A Review Article

**DOI:** 10.7759/cureus.56812

**Published:** 2024-03-24

**Authors:** Husham O Elzein

**Affiliations:** 1 Hematology, Northern Border University, Arar, SAU

**Keywords:** abo frequency, chronic lymphoid leukemia, acute lymphoid leukemia, abo discrepancy, chronic myeloid leukemia (cml), acute myeloblastic leukemia

## Abstract

The ABO system is an essential blood group in clinical transfusion medicine implicated in several human diseases. The ABO system has been investigated for over a century, with various studies exploring potential links to disease susceptibility. The study examines the possible relationship between leukemia and the distribution and the ABO blood group system discrepancy. A comprehensive review was conducted on the recommended databases to review the ABO blood groups, their association with leukemia, and the expected changes in blood groups among leukemia patients. The study highlights different kinds of leukemia, such as acute lymphocytic leukemia (ALL), acute myeloid leukemia (AML), chronic myeloid leukemia (CML), and chronic lymphocytic leukemia (CLL), their characteristics, and their relationship with ABO blood groups. The document concludes that studying ABO blood group distributions among leukemia patients showed that the most common blood group in acute leukemia is the A group, while in chronic leukemia, the O group is predominant; more studies are required. This study also confirmed an association between leukemia and ABO blood group discrepancy.

## Introduction and background

Leukemia is a hematological malignant disease characterized by the increased proliferation of abnormal leukocytes; it can be classified as either chronic or acute according to the speed of proliferation and cell line, either lymphocytic or myelocytic based on the cell of origin [1]. The ABO blood group system plays a dynamic role in blood transfusion. The composition of ABO antigens is carbohydrate base and located on the red cells' surface beside it is situated in the other cells and body fluid. In addition, these cellular antigens have been confirmed to directly affect the susceptibility to several diseases [2-4]. Several previous studies indicate that blood group antigens may play valuable and significant roles in the progression, tumor genesis, and prognosis of some diseases, particularly leukemia. Many previous studies found the ABO blood group to be associated with the risk of malignant diseases compared to normal cells, which led to an existing association between ABO antigens and chronic diseases, including leukemia; however, this association remains uncertain until now [5-9]. The present research attempts to examine the possible implications and correlation between leukemia and the distribution and discrepancy of the ABO system.

## Review

Methodology

A comprehensive review was conducted for previous studies revealing the relationship between ABO blood groups and leukemia on the recommended databases, such as Web of Science, Scopus, PubMed, and Google Scholar. Furthermore, we looked through the reference sections for related publications and reviews. Only articles in the English language were taken into consideration. Our literature covered several studies, including reviews, meta-analyses, case reports, case series, and original articles. If the full text was unavailable and the data was consistent with our study, we contacted the authors to send the full text.

The articles were carefully checked by taking out significant discussions, not only summaries, using the following keywords: ABO; ALL; AML; CML; CLL; Leukemia; Discrepancy.

Results

Acute Lymphocytic Leukemia (ALL)

This hematological malignancy is characterized by abnormal proliferation of blast cells (lymphoblast). About 80% of ALL cases occur in children; it is considered a severe disease when it appears in adults. In the United States, ALL incidences are expected at 1.6 per 100,000 populations; it becomes symptomatic quickly due to its aggressive characteristics [10-12]. ALL is developed by complicated factors affecting hematopoietic precursor cell proliferation, growth, and development, including chromosomal translocations, inversions, and point mutations [13]. Despite decades of research, the cause of ALL remains mysterious. Various factors, including radiation, toxic gases, chemicals, benzene, congenital genetic disorders, including Fanconi's anemia, Down's, neurofibromatosis, and Bloom's Syndrome, genetic disorders, and viruses, have been investigated as potential causes [14]. The relationship between ALL and ABO blood groups is controversial. According to research by Başc S et al. (2020), blood type O is the most prevalent. Alavi S et al. (2006) found 56.5% (95% CI: 45.8-67.1) more patients with the O blood group in the ALL category. Also, Vadivelu et al (2004). Investigated 522 ALL cases, and he found 14.3% (95% CI: 3.2-25.2) of patients with O blood group in another study done by Tavasolian et al. (2014) reported that ABO blood group frequency among 234 patients with ALL was 128(O), 82(A), 59(B), and 24(AB), while Sakić et al. found that 40.9% of ALL children with blood type O, 37% with blood group A, 16% with blood group B, and 6.5% with blood type AB. According to Jackson N et al.'s study, the O blood group predominated in 90 cases. Also, Martínez et al. (2019) identified 102 patients with ALL found to be with blood type O. Abd-Allateef et al. (2021) study emphasized that blood group O(+ve) is a risk factor for ALL development in young people [15-22], while Ghali HH et al. (2017), Sahin D et al. (2019), Abdulkareem et al. (2020), and Fatih KA et al. (2018) [23-26] suggested that the blood group frequency might be similar among ALL Patients and healthy individuals.

*Acute Myeloid Leukemia (AML)* 

It is a malignant disorder distinguished by the increased clonal proliferation of myeloid blasts in the bone marrow and peripheral circulation, which infiltrate other tissues. AML is considered the most predominant type of acute leukemia; most patients affected are adults. AML accounts for the most significant mortality rates annually in the United States [27]. Şahin D et al. (2020) and Alavi S et al. (2005) [28,22] mentioned in their study that we found that the most common blood group was the A+ study by Fatih KA et al. (2018). Vasan SK et al. (2016) study stated that most AML cases are classified as A [26,29]. Vadivelu et al. (2004) conducted a hospital-based retrospective study of children aged up to 12 years. There were 116 cases of acute myeloid leukemia, and Blood group information was recorded. A higher proportion of individuals are in the O blood group [16]. Siegel et al. (2012) reported in their study that the frequency of ABO and Rh (D) blood groups in 362 cases of acute myeloid leukemia (AML) was found to be 39.9% A Rh(D)+ [27]. Garba et al. (2022) found that the ABO antigenic frequency in AML varies from that of healthy nonleukemic people. AML patients had a distribution of 12 (45.0%) A antigens, 11 (44.0%) O antigens, and 2 (8.0%) B antigens, with no AB antigen [30]. Zand et al. (2010) found that the occurrence of A blood type was more prevalent in AML patients [31]. According to Allahyari et al. (2016), Out of 92 patients with AML, O+ (30.4%) was the most frequent blood group, followed by A+ (28.2%) and B+ (26.1) [32]. Hama HA et al. (2022) conducted a study in Iraq (AML), with 316 males and 217 females. The analysis revealed that the ABO blood group frequencies were: 186 (34.9%) O, 156 (29.3%) A, 131 (24.6%) B, and 30 (5.6%) AB [33]. Kumar et al.'s (2020) study included 77 cases of AML, which is 22 (28.6%) of the total cases of leukemia in the study. The research revealed that B-positive was the most frequent blood type [35]. Schiffer et al. (2017), in a further study, confirmed that there was no substantial link between AML and the patients' ABO blood type, but AML patients with the O blood type are presumably more liable to hematological malignancies [35]. *Chronic Myeloid Leukemia (CML)*

Chronic myelogenous leukemia (CML) is one of the malignant myeloproliferative disorder groups defined by the uncontrolled proliferation of mature and pre-mature myeloid cells. It starts in the bone marrow and circulates in the peripheral blood. A reciprocal translocation causes CML, t (9;22), known as the Philadelphia (Ph) chromosome. This fusion gene produces BCR-ABL1, a constitutively active tyrosine kinase, which induces a cytokine-independent proliferation of CML cells of the ABO blood group in CML cells [36]. Several studies have revealed a link between the ABO blood group and CML. One of these studies was conducted by Shahzad et al. (2013) [36], which revealed a higher association between CML and blood group B. Another study was conducted in Iraq by Adhiah AH et al. in 2008 and 2010, and Novaretti MC et al. found that most CML patients have an O blood group [37,38]. Also, in the study by Erdemir M et al. (2023), the O blood group was the most common in CML patients [39]. Some studies, such as Fatih KA et al. (2018) [26], identified that blood group A was the predominant blood group among patients with CML. A Yadav S study (2018) found that blood Group B was the most common [40]. In their research, Meher et al. (2022) found that 45 (45%) patients had a B+ blood group [41]. Salih BS et al. (2021) studied forty-five cases of CML. The study's findings indicate that blood group O (44%) predominates in the CML patients, subsequently followed by blood types A (22%), B (18%), and AB (16%) [42]. Janardhana et al. (1991) observed a corresponding conclusion that CML was frequent in patients with O blood groups [43]. *Chronic Lymphoid Leukemia (CLL)*

Chronic lymphocytic leukemia (CLL) is a monoclonal, malignant condition characterized by accelerated proliferation and accumulation of mature and functionally incompetent lymphocytes. CLL is the most frequent among adults in Western countries [43]. Most patients live for at least five years, while some patients pass away several years after diagnosis, mainly due to complications from CLL [44]. The distribution of ABO in CLL was revealed by Erdemir M et al. (2023) and Fatih KA et al. (2018) [39,26]. Studies show that the A blood group is the most frequent in CLL cases. Hallek M et al. (2021) [44] reported that the A blood group was the most common in CLL patients in Turkey. But Novaretti et al. (2008) and Fatih KA et al. (2018) [38,26] detected that the most common blood group was the O blood group in CLL patients. However, a Farhud DD et al.(1995) study in Iran showed that the O blood group is the most detected in CLL patients [45]. Janardhana et al. (1991) reported that Chronic lymphocytic leukemia was more prevalent among non-O blood type patients, but the result was insignificant [46]. Hyman et al. (1963). determined the presence of blood group A in individuals with eighteen out of the 21 patients with chronic lymphocytic leukemia [47]. The findings of the review are summarised in (Table 1).

**Table 1:** Frequency of ABO blood group in leukemia patients ALL: Acute lymphocytic leukemia; AML: Acute Myeloid Leukemia; CML: Chronic Myeloid Leukemia; CLL: Chronic lymphocytic leukemia

Leukemia	Finding	Reference
ALL	The most frequent blood group in ALL was (O).	[15-22]
	ALL patients and healthy individuals may have similar ABO blood groups.	[23-27]
AML	The most prevalent blood group in AML was (A) positive.	[27-31]
	Blood Group (O) was frequent.	[16,32,33,42,43]
CML	Most of the CML patients were in the blood group (O).	[37-39]
	Blood group (A) is the most frequent type.	[26]
	Blood Group (B) was the most frequent.	[36,40,41]
CLL	The most prevalent blood type in CLL patients was (A).	[26,39,44,47]
	Most of the patients are blood group (O).	[26,38,45]

Blood Group Discrepancy in Leukemia

ABO blood group can be changed to another group in rare cases; it has been commonly detected in patients with malignant disorders, particularly hematologic malignancies, predominantly malignant diseases in which the myeloid lineage takes part [48]. Because ABO antigens are not limited to red cells and are widely expressed in several human cells and tissues. This change can cause a temporary discrepancy in the blood group and can return to the actual blood group after remission from leukemia [49-51]. The proposed cause of these changes in blood grouping is due to, in some cases of leukemia, the ABO antigens on the red cell showing an epigenetic modification of the ABO gene in RBCs by leukemic cells, leading to its suppression and subsequent discrepancy in the blood grouping.

According to earlier research, two pathways account for the loss or weakening of ABH antigens in leukemia. First, the loss or decline of A or B antigens and the rise in antigen H are caused by the inactivity of A and B transferase. Secondly, these changes in the ABH antigen may be due to the loss or decreased expression of ABH transferees; this is because the ABO gene is situated on chromosomal band 9q34; the H (FUT1) gene, in contrast, is located on chromosomal band 19q13. In myeloid leukemia, there is a recurrent deletion of the 9q23-31 region, which in some cases may extend to 9q34 and cause diminished expression, eventually followed by a change in the blood group [52,53]. Some research suggested that the blood group changes during leukemia are caused by hematologic malignancy, this was first reported by van Loghem et al. who described fragile A antigen expression on the red cells of a patient with (AML), who had previously shown consistent A antigen expression [54]. When comparing AB antigen to the expression in healthy controls, the expression of A, B, or H antigens in leukemia patients had decreased between 17% and 37%. Reduced expression of A or B antigens was seen in patients with myeloid malignancies. Compared to healthy controls of the same ABO genotype, blood type (O) patients' H antigen levels were lowered by 55% and 21%, respectively [55]. Fateen T et al. (2022) found blood group discrepancies in 5 out of 10 cases (2.5%), and this change should be resolved by doing both forward and reverse grouping to the patients [56]. Also, blood group discrepancy in the ABO blood group was detected by Grujić J et al. (2022) [57]; during the blood grouping of a 27-year-old female patient with AML. The blood group indicated it was (O). Still, the reverse blood group test reading showed no reactions with A1 and B cells, which is not logical when reviewing the patient's records and genotyping determined blood group (A). The (A+) blood group was identified without discrepancy during testing after the patient entered the remission stage of the disease. According to Bianco T et al. (2001) [48], changes in ABH antigens have become common in myeloid malignancy recently; 55% (16/29) of patients with blood group (A, B, or AB) had red cells with lesser expression of A or B antigens when compared with 127 healthy people with blood group (A, B, or AB) who showed no changes. In addition, loss of H antigen was detected in 21% (6/28) of the group (O) patients but not in any of the 51 healthy group (O) persons. Maria Shafiq et al. (2015) also report a rare cause of ABO discrepancy in an elderly female with CML, and her ABO group showed no reaction with Anti-A or Anti-B in the forward blood grouping test [58]. In the reverse typing, there was a strong reaction with B cells only, while there was no reaction with A cells. Ting SC et al. (2016) reported two cases of (AML) patients who revealed a change in the ABO blood group test using forward and reverse blood grouping methods; the study concluded that the reduction in antigen A expression on the red cell membrane was what caused the change in these cases, which is supposed to be secondary to AML [59]. In Korea, Cho et al. (2011) concluded that antigens A, B, and H can be diminished or eliminated, particularly in people who have hematologic malignancies. They report a 42-year-old female with AML who had lost A antigen on her red cells. Even after three sessions of chemotherapy, she still had leukemia. Her direct blood group revealed a discrepancy: the cell type was O, but the reverse grouping was A [60]. Mishra D et al.'s (2019) investigation revealed that the blood group was O in the forward grouping; however, in the reverse grouping, only B cells agglutinated. Forward grouping was repeated to confirm the blood group. A blood type was suspected based on reverse grouping and the reaction of the patient's red cells with anti-AB, despite the lack of response with anti-A even after 1 hour of incubation at 4°C [61]. The cell grouping of a leukemia patient was also studied by Chenna et al. (2019) and revealed (O), and the serum grouping result showed B, which is a discrepancy to be resolved [62]. The original patient's blood group was confirmed to be B. The study of Radhakrishnan et al. (2016) involved an AML patient who was (O+) at the time of presentation [63]. His disease was in remission after two sessions of induction chemotherapy, but his blood type changed to (A+). In the Fateen T et al. (2022) [26] study, 157 (78.5%) cases of ALL and 43 (21.5%) cases of AML patients were included. There was a change in the blood group in five cases (2.5%). A comprehensive assessment, such as bacterial infection, is essential to address the role of blood type discrepancies and manage patients' blood transfusion requirements [64].

## Conclusions

Studies on the association between blood group types and leukemia are scarce, and the results are controversial. So according to previous studies, the current study found that the most common blood group among patients with acute leukemia (ALL, AML) is blood group A, while some studies found no difference in the distribution of the ABO group between acute leukemia and healthy individuals. Previous studies confirmed that CLL patients' most common blood group is the A group, but other studies show that blood group O is predominant. Regarding CML cases, the studies found that the most predominant blood group in CML patients is the O and B blood groups. So, according to these findings, we can't say whether blood groups can be used as an epidemiological marker for these malignancies. As a result, studies with blood groups should be continued with different study designs. This study also confirmed an association between leukemia and ABO blood group changes. The suggested origin for these blood group discrepancies is that, in some instances of leukemia, the ABO antigens on the red cell exhibit an epigenetic change of the ABO gene in RBCs due to leukemic cells, resulting in its suppression and subsequent shift in the blood grouping. In hematologic malignancies, the blood group antigens that undergo ABO antigen change are reverted to the original blood group after disease remission.
